# Flexibility in Wrestlers, Taekwondoists, and Non-Athletes During the Developmental Ages: The Effects of Sport, Age, and Sex

**DOI:** 10.3390/jfmk11010057

**Published:** 2026-01-29

**Authors:** Vassilis Gerodimos, Nikolaos Tsiakaras, Konstantina Karatrantou

**Affiliations:** Department of Physical Education and Sports Science, University of Thessaly, 42100 Trikala, Greece; ntsiakar@uth.gr (N.T.); kokaratr@uth.gr (K.K.)

**Keywords:** testing, range of motion, sit and reach, back scratch, wrestling, taekwondo

## Abstract

**Objectives:** Flexibility may be influenced by several factors, including age, sex, and physical activity or sport. This study simultaneously investigated the effect of sport (wrestling vs. taekwondo vs. no participation in sports activities), age (children vs. adolescents), and sex (boys vs. girls) on lower and upper body flexibility during the developmental ages. **Methods**: A total of 120 wrestlers, 120 taekwondoists, and 120 non-athletes (60 boys: 30 children and 30 adolescents; 60 girls: 30 children and 30 adolescents, per group) participated in the present study and performed two flexibility tests (sit and reach, back scratch). **Results**: ANOVAs and ANCOVAs (using anthropometric characteristics as covariates) analyses showed greater (*p* = 0.000–0.005) flexibility values in wrestlers and taekwondoists than non-athletes (except for the sit and reach in children girls, and flexibility of the right hand in children boys and girls, where no differences were observed). However, no differences (*p* = 0.672–0.992) were presented between wrestlers and taekwondoists (except for the flexibility of the left hand, where wrestlers showed greater values). Within the wrestlers and taekwondoists groups, children exhibited lower (*p* = 0.01–0.04) values than adolescents; while, in non-athletes, no age-related differences were observed (*p* = 0.263–0.995). Additionally, girls demonstrated higher values than boys, and the right hand demonstrated higher flexibility values than the left hand (*p* = 0.000–0.04). The difference between hands was greater (*p* = 0.000–0.01) in non-athletes (69.14–96.22%) vs. athletes (23.73–58.85%), taekwondoists (41.01–58.85%) vs. wrestlers (23.73–47%), and boys (44.68–96.22%) vs. girls (23.73–70.44%). **Conclusions**: It seems that engaging in wrestling and taekwondo sports affects the growth pattern of flexibility in boys and girls during the developmental ages.

## 1. Introduction

Wrestling and taekwondo are two demanding and intensive combat sports activities that require high levels of various physical abilities, such as strength and power, aerobic and anaerobic capacity, and coordination and flexibility [1,2,3,4]. Especially in wrestling, an adequate level of flexibility plays a crucial role in the effective execution of various wrestling offensive and defensive maneuvers, such as wrestling bridge, cradle, lowering of the center of gravity during a defensive stance, granby roll, etc. [5,6]. Additionally, in taekwondo, flexibility plays an important role in the effective execution of various taekwondo athletic skills [1] such as kicking techniques (i.e., roundhouse kick, side kick), low stances and forms (poomsae), etc. It should be mentioned, however, that both sports activities (wrestling and taekwondo), due to their intensive nature, demonstrate a high incidence of injuries, especially at the knee and shoulder joints [7,8,9,10,11,12]. There is evidence, however, that flexibility, except for the appropriate execution of various athletic skills, may help to prevent injuries in athletes [4]. Thus, the measurement and evaluation of lower and upper body flexibility, as well as the creation of indicative values across different sports, genders, and age categories, can assist coaches in making the appropriate training decisions both for performance enhancement and injury prevention.

Flexibility is influenced by several factors, including age, sex, sport, and anthropometric characteristics (i.e., limb length, trunk length) [13,14,15], which, however, have not been sufficiently studied regarding how they interact with each other and affect the specific ability. To date, several studies have been conducted in the international literature that evaluated flexibility and created indicative values/norms for the general pediatric population, examining the age- and/or sex-related differences in flexibility [13,14,15]. There is evidence that flexibility changes during the developmental ages due to the different growth rates of the skeleton and myotendinous structures [15]; however, the pattern of change in flexibility may differ between trained and untrained populations across the developmental ages [16]. Furthermore, previous researchers indicated that the age of 6–10 years is considered the optimal timeframe for the development of flexibility [17,18]. Nevertheless, there is minimal information regarding flexibility for children aged 10 years old and under, particularly in the wrestling and taekwondo athletic population. It should also be mentioned that few previous studies, in wrestlers or taekwondoists, have examined the age- [6,19,20,21], sex- [21,22], or sport-related [22,23,24] differences in flexibility. No previous study has concurrently investigated the combined effect of sport, age, and sex on flexibility performance during the developmental ages.

Regarding the age effect, previous studies in young wrestlers were focused only on male adolescent athletes (from 13 to 18 years old, depending on the study), demonstrating conflicting results, either no age-related differences among groups, or significant age-related differences in flexibility [6,19,20], while there is no information in the female or children’s wrestling athletic population. Additionally, most of the above studies in wrestlers (except the study of Evans et al.) [6] focused only on lower body flexibility (using the sit and reach test), whereas the information on upper body flexibility is limited. In taekwondo, the only study [21] that focused on age-related differences (using various age-groups from 7 to 9 years old to +33 years old) in male and female taekwondo athletes, also reported conflicting results regarding lower body flexibility (using the sit and reach test), whereas there is no information regarding upper body flexibility.

In terms of the sex-related effect on flexibility, the existing information in the international literature is very limited in young wrestlers or taekwondoists. Two previous studies that examined the sex-related differences in young wrestlers or taekwondoists demonstrated greater flexibility values (using the sit and reach test) in female compared to male athletes [21,22], whilst, to the best of our knowledge, no previous study either in wrestling or in taekwondo has investigated the sex-related differences in upper body flexibility during the developmental ages.

Finally, previous studies in young wrestlers and taekwondoists that examined the sport-related effect only on lower body flexibility (using the sit and reach test) reported ambivalent results. Indeed, Longo et al. [22] showed greater flexibility values (using the sit and reach test) in adolescent (11.9–14.9 years old; girls and boys) taekwondo compared to wrestling athletes, while no differences were observed between judo and taekwondo or judo and wrestling athletes. On the other hand, Marques et al. [23] did not report significant differences between male adolescent judo and wrestling athletes, and Adanan et al. [24] reported no sport effect on flexibility (using the sit and reach test) of male adolescent taekwondo athletes, wushu athletes, and non-athletes.

Taking all the above into consideration, the main objective of this study was to assess lower (using the sit and reach test) and upper body (using the back scratch test) flexibility levels and create indicative values, investigating simultaneously the effects of sport (wrestling vs. taekwondo vs. no-participation in sports activities), age (children vs. adolescents), and sex (boys vs. girls) on the above fitness parameter. Additionally, the secondary objectives of this study were to examine the sport-, age-, and sex-related differences in anthropometric characteristics (body mass, body height, sitting height, leg length, arm length) as well as to examine possible relationships between flexibility tests and anthropometric characteristics.

## 2. Materials and Methods

### 2.1. Sample

**Figure 1:**
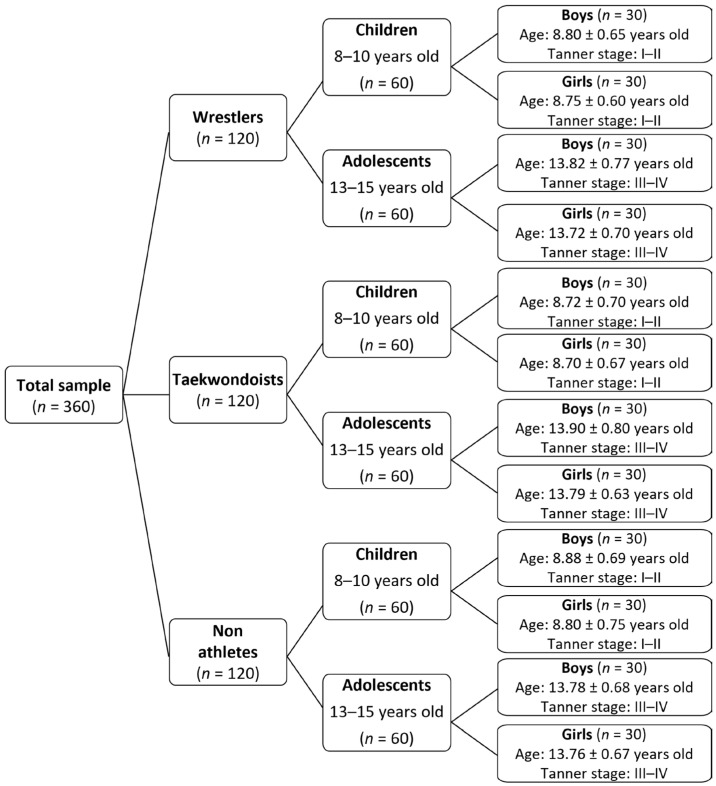
Sample of the present study.

Before the initiation of the study, a statistical power analysis (using GPower 3.1 software, developed by Faul, Erdfelder, Lang, and Buchner, Germany) indicated that a total number of 251 participants (20 participants at each sub-group) would yield adequate power 0.95 (a err prob 0.05; effect size 0.25). Thus, the final total sample (*n* = 360) of the present study (Figure 1) consisted of 120 wrestlers, 120 taekwondoists, and 120 non-athletes (30 participants in each sub-group).

The participants of the present research had to meet certain conditions to take part in the present study. Firstly, the wrestlers and taekwondoists (a) should have at least one year of training experience in sport (wrestling or taekwondo) and training frequency at least 3 times per week, and (b) except for the training of the sport (wrestling or taekwondo), did not perform any specific flexibility training program. On the other hand, non-athletes should not systematically participate in any form of exercise for physical fitness enhancement. Furthermore, the participants (boys and girls) should be children 8–10 years old (Tanner stage I–II; with no menstrual period in girls) or adolescents 13–15 years old (Tanner stage III–IV; with menstrual period in girls). Additionally, the participants should be healthy (without any chronic disease) and without injury in the upper and lower limbs for at least 6 months before the start of the study.

### 2.2. Measures

#### 2.2.1. Biological Age

Biological age was self-estimated through Tanner’s sexual maturation stages (I, II, III, IV, and V) and was determined according to pubic hair development in both boys and girls [25]. Additionally, in girls, we asked if they had a menstrual period to discriminate between children and adolescents.

#### 2.2.2. Anthropometric Characteristics

- Body Mass and Standing Body Height: The measurements of body mass and standing body height were carried out according to the guidelines of Bar-Or [26], using a calibrated physician’s scale (Seca model 755, Seca, Hamburg, Germany) and a telescopic height rod (Seca model 220, Seca, Hamburg, Germany), respectively. The measurements were taken with an accuracy of 0.5 kg and 0.1 cm, respectively, and were repeated twice.
- Sitting Body Height: The participant sat upright on a standardized box looking straight ahead, with their feet flat on the floor and their hands on their thighs. From this position, the stature is measured from the top of the vertex to the base of the sitting surface [26]. The measurement was conducted with an accuracy of 0.1 cm and was repeated twice.
- Leg Length: Following the measurements of standing body height and sitting body height, the leg length was estimated using the following equation: Leg length = Standing body height (in cm)-Sitting body height (in cm) [15].
- Total Arm Length: The participant stood upright, with their body weight evenly distributed between both feet and their arms hanging freely at the sides. The participant’s arm and hand were extended, allowing the arm to gain its full length. The measurement was performed (in both hands) from the acromion to the tip of the middle finger (dactylion) [27], using a measuring tape. The measurement was conducted with an accuracy of 0.1 cm and was repeated twice on each hand.

#### 2.2.3. Flexibility

Lower and upper body flexibility was assessed using (a) the sit and reach test and (b) the back scratch test (also called zipper test or shoulder stretch test). A previous study [28] showed high intrasession and intersession reliability of sit and reach and back scratch tests for boys and girls athletes (wrestlers and taekwondoists) as well as non-athletes during the developmental ages (children and adolescents), with ICC values ranging from 0.988 to 0.999.

- Sit and Reach Test: The participants sat barefoot on a gymnastic mat with their knees extended and their feet touching the inner surface of a Flex-Tester box (Novel Products Inc., Rockton, IL, USA). Starting from the above position, the participants should lean forward slowly with both hands, as much as possible, without bending their knees and maintaining their final position for 2 s [29]. The participants performed three attempts (with 15 s rest between them), and the best score (in cm) was considered for analysis.
- Back Scratch Test: The measurement of the back scratch test was carried out according to the guidelines of Corbin et al. [30], using a measuring tape. The participant stood upright with their legs together and extended, and their arms placed behind their back, trying to bring their middle fingers of the two hands closer and maintain the final position for 2 s. The participants performed three attempts for each hand (rest between attempts: 30 s), and the best score (in cm) for each hand was used for further analysis. It should be mentioned that the distance between the tips of the middle fingers of the two hands was recorded as the result of the measurement. If the fingertips of the two hands (i) did not touch each other, the score was negative; (ii) touched each other, then the score was zero; and (iii) overlapped each other, then the score was positive.

### 2.3. Design and Procedures

The current research was conducted according to the Declaration of Helsinki and approved by the Ethics Committee of the University of Thessaly. Before the start of the study, the participant’s parents (a) were informed about the experimental procedures and (b) signed an informed consent form. Thereafter, two to three days before the start of the study, the participants were informed and familiarized with the testing procedures. On the same day, all the participants completed a personal information form that included their training background (training age, training frequency) in wrestling or taekwondo.

Following familiarization, the participants came to the Training and Physical Conditioning Lab of the Department of Physical Education and Sport Sciences of the University of Thessaly for the measurements. The sequence of measurements was (1) biological age, (2) body mass, (3) body height, (4) sitting body height, (5) total arm length (right hand, left hand), (6) sit and reach test, and (7) back scratch test. Before the commencement of the two flexibility tests, the participants in each group performed a standardized 10 min warm-up protocol (4 min of static run, 4 min of static and dynamic stretching, and 2 min of preliminary submaximal trials). All measurements were performed by the same investigator under the same conditions. The participants were asked to follow their normal diet for two days before the study, to abstain from intense exercise activity for 24 h before the study, and to have sufficient rest the night before the study.

### 2.4. Statistical Analysis

IBM SPSS Statistics v.31 software (IBM Corporation, Armonk, NY, USA) was used to analyze the data. The effects of sport (wrestlers, taekwondoists, non-athletes), age (children, adolescents), and sex (boys and girls) on anthropometric characteristics, as well as on sit and reach values, were analyzed using three-way ANOVAs. An analysis of covariance (ANCOVA), using the variables “sitting height” and “leg length” as covariates, was conducted to examine the sport-, age-, and sex-related differences in sit and reach values. A 4-way ANOVA was used to examine the effects of sport (wrestlers, taekwondoists, non-athletes), age (children, adolescents), sex (boys and girls), and hand (right and left) on back scratch values, with repeated measures on the “hand” factor. An analysis of covariance (ANCOVA), using the variables “total arm length of right hand” and “total arm length of left hand” as covariates, was conducted to examine the sport-, age-, sex-, and hand-related differences in back scratch values. Moreover, a three-way ANOVA was used to examine the effects of sport, age, and sex on the percentage difference in flexibility between the two hands. Significant ANOVAs and ANCOVAs were followed by Sidak’s multiple comparisons to locate the significantly different means. The effect sizes were calculated using the *cohen d* equation: d = difference between means/pooled SD, where the small, medium, and large effects were reflected in values 0.20, 0.60, and 0.80, respectively [31]. Additionally, Pearson’s correlations were used to determine the relationships of flexibility tests (sit and reach, back scratch) with anthropometric characteristics (body mass, body height, sitting height, leg length, arm length) in the total sample of the present study. The level of significance was set at *p* < 0.05.

## 3. Results

### 3.1. Anthropometric Characteristics: Effect of Sport, Age, and Sex

Analysis of variance revealed no significant three-way interaction effect (sport × age × sex) or main effect of “sport” factor on body mass, standing body height, and leg length (*p* = 0.45–0.86), but showed significant two-way interaction (age × sex; *p* = 0.02) as well as significant main effects of “age” and “sex” (*p* = 0.001–0.000) factors (Table 1). Regarding the age effect, within the wrestler, taekwondo, and non-athlete groups (in boys and girls), adolescents exhibited higher (*p* = 0.001–0.000; *cohen d* = 0.85–1.30) body mass, standing body height, and leg length values than children. Concerning the sex effect, within the wrestlers, taekwondo, and non-athlete groups of adolescents, boys demonstrated higher body mass, standing body height, and leg length values than girls (*p* = 0.001; *cohen d* = 0.80–1.20), while, in the children’s groups, no differences were observed between boys and girls (*p* = 0.35–0.70). It should be mentioned that pairwise comparisons revealed no significant differences in body mass, standing body height, and leg length values among wrestlers, taekwondoists, and non-athletes (*p* = 0.65–0.86), irrespective of age and sex.

Furthermore, analysis of variance revealed no significant three-way interaction effect (sport × age × sex) or main effect of “sport” and “sex” factors on sitting height and arm length of right and left hands (*p* = 0.60–0.90), but showed significant main effect of “age” factor (*p* = 0.001–0.000; Table 1). Concerning the age effect, within the wrestler, taekwondo, and control groups (in boys and girls), adolescents exhibited higher (*p* = 0.001–0.000; *cohen d* = 0.70–1.10) sitting height and arm length values than children. However, pairwise comparisons revealed no significant differences among wrestlers, taekwondoists, and non-athletes (*p* = 0.55–0.90), irrespective of age and sex, as well as between boys and girls (*p* = 0.60–0.73), irrespective of sport and age.

### 3.2. Training Characteristics: Effect of Sport, Age, and Sex

Analysis of variance revealed no significant three-way interaction effect (sport × age × sex) or main effects of “sport” and “sex” factors on training age and frequency (*p* = 0.65–0.87), but showed a significant main effect of “age” factor (*p* = 0.001; Table 1). Concerning the age effect, within the wrestler and taekwondo groups (in boys and girls), adolescents exhibited higher (*p* = 0.001; *cohen d* = 0.73–1.35) training age and frequency values than children. However, it should be mentioned that pairwise comparisons revealed no significant differences between wrestlers and taekwondoists as well as between boys and girls (*p* = 0.60–0.88).

### 3.3. Correlation Between Anthropometric Characteristics and Flexibility Tests

Pearson’s correlation analysis in the total sample revealed significant correlation of sitting height (*r* = 0.20 small positive; *p* = 0.04) and leg length (*r* = −0.25 small negative; *p* = 0.04) with sit and reach test, while no significant correlation (*r* = −0.008–0.024; *p* = 0.654–0.876) was observed between sit and reach test and other anthropometric characteristics (body height, body mass, arm length). Moreover, Pearson’s correlation analysis in the total sample demonstrated significant correlation of right arm length (*r* = 0.24 small positive; *p* = 0.04) and left arm length (*r* = 0.27 small positive; *p* = 0.04) with back scratch test in the right and left hands, respectively, while no significant correlation (*r* = −0.018–0.028; *p* = 0.554–0.775) was observed between back scratch test and other anthropometric characteristics (body height, body mass, sitting height, and leg length).

### 3.4. Flexibility: Effect of Sport, Age, and Sex

#### 3.4.1. Sit and Reach Test

Analysis of variance revealed a significant three-way interaction effect (sport × age × sex) on the sit and reach test (*p* = 0.001; Table 2). More specifically, in the age group of children, boys wrestling and taekwondo athletes showed greater sit and reach values than boys non-athletes (*p* = 0.001; *cohen d* = 1.01–1.23), whereas, in girls, no differences were observed among wrestling, taekwondo, and non-athletes (*p* = 0.57–0.87). On the other hand, at the age group of adolescents (in boys and girls), wrestling and taekwondo athletes exhibited greater sit and reach values than non-athlete boys (*p* = 0.000; *cohen d* = 1.60–1.75) and girls (*p* = 0.000; *cohen d* = 0.45–0.74). It should be mentioned that pairwise comparisons revealed no significant differences in sit and reach values between wrestling and taekwondo athletes (*p* = 0.76), irrespective of age and sex. Regarding the age effect, within the wrestlers’ and the taekwondo groups (in boys and girls), children exhibited lower (*p* = 0.01–0.04; *cohen d* = 0.37–0.84) sit and reach values than adolescents, while within the non-athlete groups (in boys and girls), no differences were observed between children and adolescents (*p* = 0.82–0.85). Concerning the sex effect, within the wrestlers, the taekwondo and the non-athlete groups (in children and adolescents), girls demonstrated higher sit and reach values than boys (*p* = 0.001–0.000; *cohen d* = 0.47–1.40).

**Figure 2:**
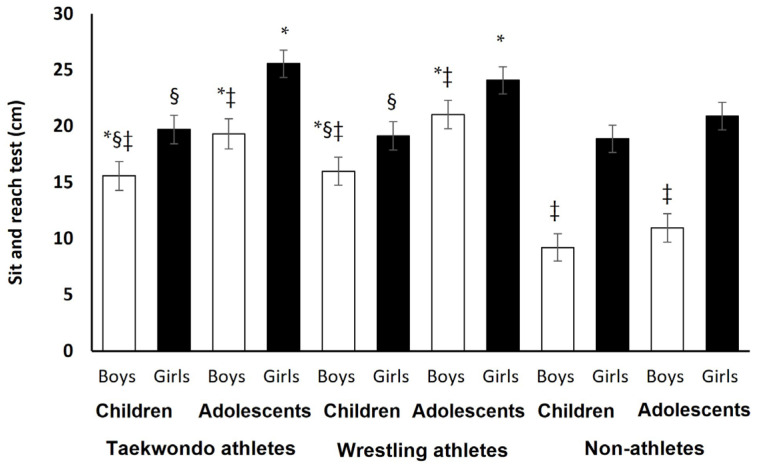
Sit and reach values per sport, age, and sex. Mean covariates appearing in the model are evaluated at the following values: leg length = 71.90, sitting height = 78.64. Where * *p* = 0.001–0.000 between athletes (wrestlers or taekwondoists) and non-athletes; § *p* = 0.01–0.04 between children and adolescents; ‡ *p* = 0.001–0.000 between boys and girls.

According to the ANCOVA analysis, after the statistically significant adjustment of the covariates (sitting height and leg length; *p* = 0.04), the results continued to show significant three-way interaction effect (sport × age × sex) on the sit and reach test (*p* = 0.001; *cohen d* = 0.40–1.70; Figure 2), as we analytically presented above.

#### 3.4.2. Back Scratch Test

Analysis of variance revealed a significant four-way interaction effect (sport × age × sex × hand) on the back scratch test of the right and left hands (*p* = 0.001; Table 3). More specifically, at the age group of children, boys and girls wrestlers and taekwondoists showed greater back scratch values in the left hand than boys and girls non-athletes (*p* = 0.001; *cohen d* = 0.35–1.10), while no differences were observed among wrestlers, taekwondoists, and non-athletes in the right hand (*p* = 0.50–0.60). On the other hand, at the age group of adolescents (in boys and girls), wrestlers and taekwondoists exhibited greater back scratch values than boys and girls non-athletes (*p* = 0.001–0.000; *cohen d* = 0.45–1.15) in both hands. Additionally, it should be mentioned that pairwise comparisons revealed greater back scratch values of the left hand in wrestlers compared to taekwondoists (*p* = 0.03), irrespective of age and sex.

Regarding the age effect, within the wrestler and the taekwondo groups (in boys and girls), children exhibited lower (*p* = 0.001; *cohen d* = 0.80–1.15) back scratch values (in right and left hands) than adolescents. While within the control groups (in boys and girls), no differences were observed between children and adolescents in right and left hands (*p* = 0.68–0.79). Concerning the sex effect, within the wrestlers, taekwondo, and non-athlete groups (in children and adolescents), girls demonstrated higher back scratch values than boys (*p* = 0.001–0.000; *cohen d* = 0.27–1.25). Finally, within the wrestlers, taekwondo, and non-athlete groups, analysis of variance showed significant differences between right and left hands, where the right hand demonstrated higher back scratch values than the left hand (*p* = 0.001–0.000; *cohen d* = 0.42–0.73). It should be mentioned that the percentage difference in flexibility between hands was greater (*p* = 0.000–0.01; *cohen d* = 1–1.90) in non-athletes (69.14–96.22%) vs. wrestlers and taekwondoists (23.73–58.85%), as well as in taekwondoists (41.01–58.85%) vs. wrestlers (23.73–47%). Moreover, the percentage difference in flexibility between hands was greater (*p* = 0.01; *cohen d* = 0.90–1.70) in boys compared to girls at all sport and age groups (Table 3).

**Figure 3:**
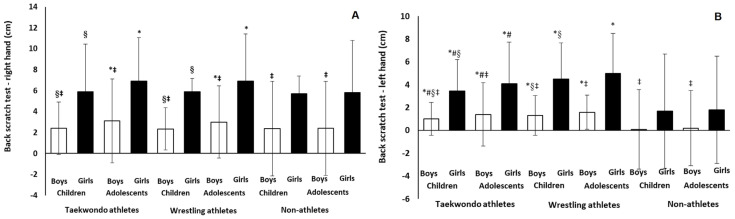
Back scratch values per sport, age, and sex for the right (**A**) and left (**B**) hands. Mean covariates appearing in the model are evaluated at the following values: arm length of the right hand = 65.57, arm length of the left hand = 65.61. Where * *p* = 0.001 between athletes (wrestlers or taekwondoists) and non-athletes; # *p* = 0.03 between wrestlers and taekwondoists; § *p* = 0.001–0.000 between children and adolescents; ‡ *p* = 0.001–0.000 between boys and girls.

According to the ANCOVA analysis, after the statistically significant adjustment of the covariates (total length of the right arm and total length of the left arm; *p* = 0.04), the results continued to show significant four-way interaction effect (sport × age × sex × hand) on back scratch test of the right and left hands (*p* = 0.001; *cohen d* = 0.50–1.80; Figure 3), as we analytically presented above.

## 4. Discussion

This is the first study that concurrently investigated and compared the sport-, age-, and sex-related differences in flexibility (lower and upper body) among wrestlers, taekwondoists, and non-athletes during the developmental ages. The main results of the present study indicated that (a) the participation in wrestling and taekwondo sports affects the development pattern of flexibility in boys and girls during the developmental ages, (b) the flexibility of lower and upper body is influenced by sex and age, (c) the tested hand affects the performance in back scratch test, and (d) different anthropometric characteristics show significant low correlation with the performance in flexibility tests (sit and reach, back scratch); however this correlation did not affect the sport-, age-, and sex-related differences in flexibility.

### 4.1. Age- and Sport-Related Differences

The findings regarding the age-related differences in flexibility are contradictory, especially those concerning whether there is a decrease in the range of motion during the peak height velocity (PHV) phase in children and adolescents [15]. It is generally accepted that children are more flexible than adults [14], while flexibility levels have a tendency to plateau or even decrease at the time of the adolescent growth spurt and into adulthood [15]. This evidence is reinforced by the results of the present study in non-athletes, where no significant changes in flexibility (sit and reach test, back scratch test) were observed between children and adolescents. Differences in muscle and tendon morphology, as well as neurophysiological alterations between children and adolescents, may reinforce the occurrence of a plateau or decline in flexibility [32,33,34,35]. In more detail, during puberty, the faster growth of bones compared to muscles can cause reduced muscle-tendon extensibility and substantial limitations on range of motion [36,37]. Additionally, the increase in hormone levels (e.g., testosterone) [38] during puberty may affect tendon stiffness and, consequently, range of motion, especially in boys [39]. On the other hand, other studies showed that flexibility performance has been positively associated with chronological age in children and adolescents [40,41,42]. While there are also studies that have identified inconsistent results regarding the effect of age on flexibility during the developmental ages. For example, a prospective study of Baquet et al. [43] in children and adolescents (11–16 years old) demonstrated a slight decrease in flexibility with increasing age in boys, whereas the flexibility performance increased in girls.

It seems that engagement or non-engagement in sports activities may influence the developmental pattern of flexibility in children and adolescents. In more detail, a previous study of Germain and Blair [16] reported that after the age of 10 years old, there was a decline of flexibility (at the shoulder joint), which was more pronounced in inactive participants than in those who participated in activities involving the upper extremities, suggesting that the rate of decline in flexibility across age was less for individuals who were active. In the same context, in the present study, we also found different age-related alterations in lower and upper body flexibility between athletes (wrestlers and taekwondoists) and non-athletes during the developmental ages. Indeed, wrestlers and taekwondoists (boys and girls) showed a significant increase in flexibility with age, while non-athletes demonstrated a plateau in flexibility from childhood to adolescence. However, to the best of our knowledge, no previous study in the scientific literature has investigated the developmental pattern in flexibility between athletes (wrestlers or taekwondoists) and non-athletes to compare our results, whilst in other physical fitness indices (i.e., handgrip strength, isokinetic strength), there is some information to compare our findings. Indeed, previous studies exhibited that the pattern of absolute and relative handgrip strength development [44], as well as the isokinetic leg strength development [45], was different in young wrestlers than in non-athletes.

The engagement with wrestling or taekwondo, apart from the developmental pattern of flexibility from childhood to adolescence, may also influence the performance levels in flexibility tests. In the present study, wrestlers and taekwondoists generally showed greater sit and reach values than their non-athlete counterparts (except for the children girls), whereas no differences were observed between wrestlers and taekwondoists. According to the findings of this study, it seems that the engagement with the sports of wrestling and taekwondo equally and positively affects the performance in the sit and reach test in children and adolescents. However, in girls, the difference between athletes and non-athletes is noticeable during adolescence, while in boys, the differences in sit and reach test are evident from childhood. The few previous studies [22,23,24] that examined either differences between athletes and non-athletes or differences among various sport activities in the sit and react test demonstrated contradictory results. In accordance with the results of the present study, Adanan et al. [24], who compared the sit and reach values among young male (from 14 to 20 years old) athletes of two sport activities (taekwondo and wushu), demonstrated no significant differences. Additionally, Marques et al. [23] did not report significant differences in sit and reach values between male adolescent judo and wrestling athletes. On the other hand, Longo et al. [22] demonstrated greater sit and reach values in adolescents (11.9–14.9 years old; girls and boys) in taekwondo compared to wrestling athletes, while no differences were observed between judo and taekwondo or judo and wrestling athletes. It seems that the findings regarding the sport-related effect in the sit and reach test are limited and controversial, and, thus, future studies are necessary to draw more accurate conclusions on this topic, taking into account some factors that may affect the results, such as the training level, the maturity stage, the morphological and hormonal changes, the sample size, etc.

Regarding the back scratch test, no earlier study has investigated the possible differences among wrestlers, taekwondoists, and non-athletes to compare our results. Our findings demonstrated that athletes (wrestlers and taekwondoists) showed greater back scratch test values than non-athletes and lower lateral asymmetries between right and left hands. Specifically, in non-athletes, there are significant differences between the two hands (where the right hand showed greater values than the left hand), a finding which is in line with a previous study in untrained children and adolescents [13]. Previous studies, which found significant differences in flexibility between the two hands, hypothesized that this may be attributed to the repeated involvement of one limb in activities of daily living (ADLs) such as self-grooming activities like combing, putting on a shirt, buttoning a shirt, eating, and lifting objects [46]. The engagement in sport activities and the specific symmetrical training of both hands may positively influence lateral asymmetries (reducing the percentage differences between hands). This notion is strengthened by the results of the present study, where wrestlers showed lower flexibility asymmetries than taekwondoists. Therefore, it seems that the participation in sports activities and especially those that more symmetrically train both hands (e.g., wrestling) is an effective strategy to reduce the lateral asymmetries in flexibility between hands during the developmental ages.

### 4.2. Sex-Related Differences

The results of the present study are in line with previous studies in young athletes of taekwondo [21,22], wrestling [22], and judo [22], as well as in untrained children and/or adolescents who observed greater sit and reach values in girls compared to boys [43,47,48,49]. Concerning the upper body flexibility, our findings showed significant sex-related differences in the back scratch test (right and left hands), where girls demonstrated greater values than boys. To our knowledge, no previous study in wrestlers or taekwondoists, during the developmental ages, examined the sex-related differences in upper body flexibility to compare our results. Nevertheless, one previous study [13] in untrained children and adolescents (6–17 years old) showed inconsistent results regarding the sex-related differences in the shoulder stretch test. In more detail, Castro-Piñero et al. [13] demonstrated that girls had better performance than boys in the right shoulder stretch test from 8 to 13 years old and in the left shoulder stretch test in youths aged 10 to 15 years, whereas no sex-related differences were observed in other age groups. The greater flexibility values of girls/females compared to boys/males may be attributed to various factors, such as the lower muscle mass and muscle strength of females, the geometry and morphology of females’ joints, hormonal differences, as well as differences in collagen levels [50]. Additionally, there is evidence that a part of the differences in flexibility between boys and girls may be attributed to the different growth patterns of limbs (lower or upper) and trunk length during the developmental ages [15]. For example, in girls during the phase in which the maximum growth rate in height is observed, the length of the trunk increases more than that of the legs, maybe improving their performance in the sit and reach test. In contrast, in boys, the greatest decrease in the score of the sit and reach test occurs during the maximum growth rate in height due to an increase in leg length, while later the range of motion improves due to the simultaneous increase in the length of the trunk and upper limbs [15]. However, in the present study, although there is a significantly small correlation between anthropometric characteristics and flexibility tests, the sex-related differences in flexibility were still evident when we used anthropometric characteristics as covariates.

Furthermore, in the present study, we observed significant differences in the back scratch test between the two hands, where the right hand showed greater values than the left hand. However, it should be mentioned that the percentage difference in flexibility between the two hands was greater in boys (44.68–96.22%) compared to girls (23.73–70.44%). In the same context, Castro-Piñero et al. [13] also observed differences between the right and the left sides, where both untrained boys (from 10 years old and later) and girls (from 8 years old and later) had better scores in the right shoulder stretch test than the left shoulder stretch test. The aforementioned authors mentioned that this might be due to the lateral asymmetry or dominance, which seems to become more evident at the age of 7–8 years old and later [51]. Moreover, Castro-Piñero et al. [13], in accordance with our findings, showed a greater percentage difference between right and left sides in boys (60–100%) compared to girls (50%) from 10 to 17 years old. Taking all the above into consideration, it seems that lateral asymmetries should be taken into account in upper body flexibility tests during the developmental ages in both boys and girls (using (a) specialized exercise programs for reducing possible lateral asymmetries and (b) simple advice for balanced use of both hands in daily life), and especially in boys, where the asymmetries are greater compared to girls.

Our study has some restrictions that may possibly affect the generalization of the findings. Initially, the findings of our study are limited to young (children and adolescents) wrestlers, taekwondoists, and non-athletes, and cannot be generalized to other populations with different characteristics. Forthcoming studies may possibly investigate flexibility performance and create indicative-normative values for populations of other age groups, sports (individual or team sports), maturity stages, or training levels. Additionally, our study presents indicative values for the two flexibility tests used (sit and reach and back scratch test), and, thus, our findings cannot be generalized to other muscle groups of the human body. Nevertheless, upcoming studies may possibly evaluate flexibility performance and create indicative-normative values in other muscle groups of the human body using various field or laboratory flexibility tests. Another limitation of the study is the lack of a group that performed except for the sport-specific training and flexibility training to examine and compare solely the influence of sport-specific training, as well as the combination of sport-specific training and flexibility training. Moreover, in our study, we did not evaluate and compare (among sport, age, and sex groups) any morphological or hormonal index that may affect flexibility performance. Finally, future studies with a larger sample could create norms for flexibility performance in various populations and draw more accurate findings regarding the interaction effect of sport, age, and sex on flexibility.

## 5. Conclusions

In conclusion, lower and upper body flexibility exhibited a different developmental pattern in athletes (wrestlers and taekwondoists) and non-athletes during the developmental ages, implying that there is an interaction between biological development and training responses. From childhood, there are differences in flexibility between athletes and non-athletes; however, the differences are more evident during adolescence. The engagement in sports activities, and mainly those that equally activate both hands (e.g., wrestling), limits the handedness asymmetry in upper body flexibility observed during childhood and adolescence. Finally, girls (athletes and non-athletes) demonstrate greater lower and upper body flexibility values than boys. The results of this study have important practical implication providing valuable information (using indicative values per sport, age, and sex) to coaches regarding the flexibility performance of their athletes (wrestlers and taekwondoists). Following the evaluation of results, coaches could design and implement short-term or long-term flexibility training programs aiming at further development of the specific physical fitness parameter (depending on the needs of each athlete).

## Figures and Tables

**Table 1:** Anthropometric and training characteristics of the participants per sport, age, and sex (mean ± standard deviation).

Variables	Sport	Age Group	
		Children	Adolescents
Anthropometric characteristics			
Body mass (kg)	Wrestling athletes	Boys: 37.59 ± 9.29 Girls: 36.50 ± 7.62	Boys: 57.01 ± 9.75 *# Girls: 52.13 ± 11.72 *
	Taekwondo athletes	Boys: 36.78 ± 9.38 Girls: 35.29 ± 11.90	Boys: 55.06 ± 10.33 *# Girls: 50.80 ± 7.39 *
	Non-athletes	Boys: 37.48 ± 6.68 Girls: 36.22 ± 10.86	Boys: 56.27 ± 10.76 *# Girls: 52.17 ± 7.06 *
Body height (cm)	Wrestling athletes	Boys: 138.23 ± 9.24 Girls: 138.22 ± 7.16	Boys: 162.50 ± 10.29 *# Girls: 157.97 ± 8.73 *
	Taekwondo athletes	Boys: 139.40 ± 7.18 Girls: 138.52 ± 9.87	Boys: 166.41 ± 8.96 *# Girls: 158.36 ± 9.12 *
	Non-athletes	Boys: 138.57 ± 7.86 Girls: 138.61 ± 9.93	Boys: 162.88 ± 10.64 *# Girls: 157.82 ± 8.51 *
Sitting height (cm)	Wrestling athletes	Boys: 71.88 ± 3.94 Girls: 70.14 ± 3.74	Boys: 82.95 ± 7.12 * Girls: 82.02 ± 4.47 *
	Taekwondo athletes	Boys: 71.27 ± 3.57 Girls: 70.19 ± 5.80	Boys: 82.83 ± 6.41 * Girls: 82.81 ± 5.04 *
	Non-athletes	Boys: 71.43 ± 3.58 Girls: 70.92 ± 5.38	Boys: 82.43 ± 6.71 * Girls: 82.40 ± 5.98 *
Leg length (cm)	Wrestling athletes	Boys: 66.35 ± 6.06 Girls: 68.08 ± 4.88	Boys: 79.55 ± 4.48 *# Girls: 75.95 ± 6.00 *
	Taekwondo athletes	Boys: 68.13 ± 4.13 Girls: 68.33 ± 6.64	Boys: 83.58 ± 3.71 *# Girls: 75.55 ± 5.04 *
	Non-athletes	Boys: 67.14 ± 4.74 Girls: 67.69 ± 6.36	Boys: 80.45 ± 5.21 *# Girls: 75.42 ± 6.29 *
Arm length—right (cm)	Wrestling athletes	Boys: 60.43 ± 4.08 Girls: 60.06 ± 4.31	Boys: 70.92 ± 4.67 * Girls: 70.41 ± 4.20 *
	Taekwondo athletes	Boys: 60.66 ± 5.87 Girls: 60.17 ± 4.61	Boys: 71.08 ± 3.97 * Girls: 70.32 ± 4.54 *
	Non-athletes	Boys: 60.39 ± 3.90 Girls: 60.17 ± 4.59	Boys: 71.06 ± 4.70 * Girls: 70.25 ± 3.62 *
Arm length—left (cm)	Wrestling athletes	Boys: 60.53 ± 4.04 Girls: 60.44 ± 4.29	Boys: 70.87 ± 4.81 * Girls: 70.34 ± 4.12 *
	Taekwondo athletes	Boys: 60.68 ± 5.75 Girls: 60.21 ± 4.69	Boys: 71.07 ± 3.96 * Girls: 70.32 ± 4.54 *
	Non-athletes	Boys: 60.39 ± 3.90 Girls: 60.17 ± 4.59	Boys: 71.05 ± 4.65 * Girls: 70.25 ± 3.60 *
Training characteristics			
Training age (years of training)	Wrestling athletes	Boys: 2.68 ± 1.47 Girls: 2.67 ± 1.24	Boys: 4.60 ± 1.50 * Girls: 4.43 ± 1.36 *
	Taekwondo athletes	Boys: 2.85 ± 1.43 Girls: 2.78 ± 1.34	Boys: 4.50 ± 1.25 * Girls: 4.33 ± 1.70 *
	Non-athletes	Boys: - Girls: -	Boys: - Girls: -
Training frequency (times/week)	Wrestling athletes	Boys: 3.38 ± 0.76 Girls: 3.33 ± 0.53	Boys: 4.10 ± 1.19 * Girls: 4.00 ± 0.86 *
	Taekwondo athletes	Boys: 3.37 ± 0.37 Girls: 3.35 ± 0.25	Boys: 4.03 ± 0.99 * Girls: 4.00 ± 0.97 *
	Non-athletes	Boys: - Girls: -	Boys: - Girls: -

Where * *p* = 0.001–0.000 between adolescents and children; # *p* = 0.001 between boys and girls in adolescent groups of athletes and non-athletes.

**Table 2:** Sit and reach values per sport, age, and sex (mean ± standard deviation).

Sport	Age	Sex	Mean ± SD
Taekwondo athletes	Children	Boys	16.49 ± 7.25 cm *§‡
		Girls	20.86 ± 3.89 cm §
	Adolescents	Boys	18.72 ± 4.67 cm *‡
		Girls	25.09 ± 6.13 cm *
Wrestling athletes	Children	Boys	17.10 ± 5.87 cm *§‡
		Girls	20.37 ± 5.15 cm §
	Adolescents	Boys	19.81 ± 6.98 cm *‡
		Girls	23.36 ± 8.01 cm *
Non-athletes	Children	Boys	9.86 ± 5.91 cm ‡
		Girls	19.11 ± 8.97 cm
	Adolescents	Boys	9.85 ± 5.45 cm ‡
		Girls	19.67 ± 8.57 cm

Where * *p* = 0.001–0.000 between athletes (wrestlers or taekwondoists) and non-athletes; § *p* = 0.01–0.04 between children and adolescents; ‡ *p* = 0.001–0.000 between boys vs. girls.

**Table 3:** Back scratch values per sport, age, sex, and hand (mean ± standard deviation).

Sport	Age	Sex	Right Hand	Left Hand	Difference Between Hands
Taekwondo athletes	Children	Boys	2.43 ± 2.55 cm §‡†	1.00 ± 1.45 cm *#§‡	58.85% *#‡
		Girls	5.92 ± 4.60 cm §†	3.45 ± 2.76 cm *#§	41.7% *#
	Adolescents	Boys	3.10 ± 4.55 cm *‡†	1.30 ± 2.78 cm *#‡	58% *#‡
		Girls	6.90 ± 4.18 cm *†	4.07 ± 3.68 cm *#	41.01% *#
Wrestling athletes	Children	Boys	2.35 ± 2.15 cm §‡†	1.30 ± 1.75 cm *§‡	44.68% *‡
		Girls	5.90 ± 3.50 cm §†	4.50 ± 3.15 cm *§	23.73% *
	Adolescents	Boys	3.00 ± 2.35 cm *‡†	1.59 ± 1.50 cm *‡	47% *‡
		Girls	6.93 ± 4.50 cm *†	5.00 ± 3.50 cm *	27.85% *
Non-athletes	Children	Boys	2.38 ± 4.72 cm ‡†	0.09 ± 4.75 cm ‡	96.22% ‡
		Girls	5.75 ± 7.28 cm †	1.70 ± 7.00 cm	70.44%
	Adolescents	Boys	2.40 ± 4.55 cm ‡†	0.20 ± 4.63 cm ‡	91.67% ‡
		Girls	5.80 ± 7.13 cm †	1.79 ± 7.38 cm	69.14%

Where * *p* = 0.001 between athletes (wrestlers or taekwondoists) and non-athletes; # *p* = 0.03 between wrestlers and taekwondoists; § *p* = 0.001–0.000 between children and adolescents; ‡ *p* = 0.001–0.000 between boys and girls; † *p* = 0.001–0.000 between right and left hands.

## Data Availability

The original contributions presented in this study are included in the article. Further inquiries can be directed to the corresponding author.
